# Age related vascular endothelial function following lifelong sedentariness: positive impact of cardiovascular conditioning without further improvement following low frequency high intensity interval training

**DOI:** 10.14814/phy2.12234

**Published:** 2015-01-27

**Authors:** Fergal M. Grace, Peter Herbert, John W. Ratcliffe, Karl J. New, Julien S. Baker, Nicholas F. Sculthorpe

**Affiliations:** Institute of Clinical Exercise & Health Sciences, School of Science and Sport, University of the West of Scotland, Hamilton, UK; University of Wales Trinity Saint David, CamarthenWales, UK; School of Health Sport and Professional Practice, University of South Wales, Wales, UK

**Keywords:** Aging, angiogenesis, high intensity interval training, vascular function

## Abstract

Aging is associated with diffuse impairments in vascular endothelial function and traditional aerobic exercise is known to ameliorate these changes. High intensity interval training (HIIT) is effective at improving vascular function in aging men with existing disease, but its effectiveness remains to be demonstrated in otherwise healthy sedentary aging. However, the frequency of commonly used HIIT protocols may be poorly tolerated in older cohorts. Therefore, the present study investigated the effectiveness of lower frequency HIIT (L_*f*_HIIT) on vascular function in a cohort of lifelong sedentary (SED;* n *=**22, age 62.7 ± 5.2 years) men compared with a positive control group of lifelong exercisers (LEX;* n* = 17, age 61.1 ± 5.4 years). The study consisted of three assessment phases; enrolment to the study (Phase A), following 6 weeks of conditioning exercise in SED (Phase B) and following 6 weeks of low frequency HIIT in both SED and LEX (L_*f*_HIIT; Phase C). Conditioning exercise improved FMD in SED (3.4 ± 1.5% to 4.9 ± 1.1%; *P *<**0.01) such that the difference between groups on enrolment (3.4 ± 1.5% vs. 5.3 ± 1.4%; *P *<**0.01) was abrogated. This was maintained but not further improved following L_*f*_HIIT in SED whilst FMD remained unaffected by L_*f*_HIIT in LEX. In conclusion, L_*f*_HIIT is effective at maintaining improvements in vascular function achieved during conditioning exercise in SED. L_*f*_HIIT is a well‐tolerated and effective exercise mode for reducing cardiovascular risk and maintaining but does not improve vascular function beyond that achieved by conditioning exercise in aging men, irrespective of fitness level.

## Abstract (precis)

The effects of low frequency high intensity interval training (HIIT) on vascular endothelial function in lifelong sedentary men remains currently unknown. The present study examined the impact of low frequency HIIT following conditioning exercise on low determinants of vascular endothelial function and angiogenic biomarkers in aging men compared with a positive control group of similarly aged. The major findings of this study indicate that low frequency HIIT is a well‐tolerated and effective exercise mode for reducing cardiovascular risk and maintaining but not improving endothelial function beyond that achieved by conditioning exercise in aging men, irrespective of fitness level.

## Introduction

Normal physiological aging is accompanied by diffuse alterations to vascular structure and function that contrive to increase cardiovascular risk with advancing years (Lakatta and Levy 2003a,b). Although, many of the underlying mechanisms remain to be fully elucidated, stiffening of the large elastic arteries and concomitantly impaired endothelial function play central roles in the etiology of atherogenesis and endothelial dysfunction both of which predispose older adults to the development of cardiovascular disease (Seals 2014).

The benefits of regular exercise are widely acknowledged and older adults may garner particular benefit by stemming the age associated decline in muscle mass and aerobic capacity (Chodzko‐Zajko et al. 2009a), maintenance of cognitive function (Chrysohoou et al. 2014), psychological well‐being and quality of life (Penedo and Dahn 2005). Despite the clear advantages, many older adults remain sedentary and few achieve the recommended levels of physical exertion needed to accrue these health benefits. Low levels of cardiorespiratory fitness are associated with increased risk for cardiovascular and all‐cause mortality in people of all ages, and initiation of a regular exercise regime lessens risk across the lifespan (Paffenbarger et al. 1993; Thompson et al. 2003). Moreover, given that small improvements in cardiorespiratory fitness can have a major impact on health and survival (Kodama et al. 2009; Kaminsky et al. 2013) then it stands to reason that lifelong sedentary individuals who take‐up exercise later in life stand to achieve the greatest health benefit outcomes.

Reduced endothelial nitric oxide synthase (eNOS) activity and/or impaired responsiveness to nitric oxide (NO) during advancing age, reduces endothelium‐dependent vasodilation. Endothelium‐mediated coronary vasodilation is the principal physiologic channel for the increased coronary blood flow that accompanies increased stroke work. Coupled with the concomitant dampening of responsiveness to beta‐adrenergic stimulation (Whaley et al. 1992) there is an inexorable age‐associated reduction in maximal cardiovascular function.

Over the course of the past two decades, there has been a gradual accumulation of evidence in support of the beneficial effects of exercise on arterial stiffening and vascular endothelial function. Brachial artery flow mediated dilatation (FMD), is reduced in sedentary older adults but preserved in age‐matched endurance trained athletes and lifelong exercisers (Rywik et al. 1999; Eskurza et al. 2004b, 2005; Pierce et al. 2011). These studies provide encouraging evidence for the preventative effects of aerobic exercise on vascular endothelial dysfunction.

First described by ÅStrand et al. (1960), high‐intensity interval training (HIIT) has recently re‐emerged as an effective method of improving cardiovascular function in a variety of young and adult populations. HIIT is characterized by brief, intermittent bursts of vigorous exercise, interspersed by periods of rest or low intensity recovery (Gibala et al. 2012). Furthermore, HIIT may be more effective than moderate‐intensity continuous training in healthy adult, (Helgerud et al. 2007) young obese (Tjonna et al. 2009), older heart failure (Rognmo et al. 2004), and metabolic syndrome patients (Tjonna et al. 2008). HIIT has also demonstrated the capacity to markedly improve aerobic capacity and vascular function in older post cardiac rehabilitation patients (Wisloff et al. 2007). The available HIIT intervention studies that investigate the measurement of FMD in middle aged or older adults involve patients with existing vascular pathologies including obesity (Schjerve et al. 2008), coronary artery disease (Munk et al. 2009), heart failure (Wisloff et al. 2007), hypertensives (Molmen‐Hansen et al. 2012), and post‐myocardial infarction (Moholdt et al. 2012) patients. Despite the growing evidence in support of HIIT exercise amongst diseased cohorts, the ability of this exercise mode to bring about improvements of a similar magnitude during otherwise healthy sedentary aging, remains to be demonstrated.

Although current exercise guidelines provide endorsement that exercise should be performed at a minimum of twice per week to confer improvements in cardio‐respiratory function (Chodzko‐Zajko et al. 2009b), evidence demonstrates that as few as six sessions of low volume HIIT performed over 2 weeks is sufficient to stimulate in vivo physiological remodeling and metabolic adaptation (Burgomaster et al. 2005; Gibala et al. 2006) at levels comparable with moderate intensity aerobic exercise despite a markedly lower time commitment and reduced exercise volume (Gibala and McGee 2008). Given that older individuals can take longer to recover from strenuous exercise than younger counterparts (Klein et al. 1988) and recovery from fatiguing exercise can exceed 5 days (Clarkson and Tremblay 1988; Dedrick and Clarkson 1990), HIIT protocols utilizing standard frequencies may be overly fatiguing and thus poorly tolerated in sedentary older cohorts.

Further developing our understanding of the effect of exercise mode on vascular function is imperative for therapeutic exercise prescription. Therefore, investigation of low‐frequency (once every 5 days) high intensity interval exercise training (L_*f*_HIIT) in older individuals is warranted. Moreover, the most effective prescription (frequency, intensity, time, type) to produce health benefits during advancing age in sedentary cohorts remains to be elucidated. With this in mind, the present study aimed to examine the efficacy of low frequency HIIT (L_*f*_HIIT) on vascular dependent vasodilation in sedentary aging males and compare them with an age‐matched positive control group consisting of lifelong exercisers. A further aim was to investigate potential avenues for the mediation of improvements. We hypothesized that lifelong sedentary males (SED) would demonstrate inferior vascular function when compared with lifelong exercisers (LEX) and further hypothesized that a program of supervised (L_*f*_HIIT), subsequent to conditioning exercise, would favorably affect vascular function in SED compared with LEX.

## Methods

### Ethical approval

Participants consisted of male volunteers (*n* = 47), over the age of 55 years, who had responded to recruitment posters placed in leisure centers, medical surgeries, public houses, coffee shops, and newsagents in the Camarthen district of South Wales, UK. Participants were met individually for an informal explanation of the study objectives and supplied with a participant information sheet. As a condition to study enrolment, general medical practitioners (GP's) for each potential participant were contacted and provided with a copy of the study design, protocols, and intended exercise programs. GP's were required to provide a written letter of approval for their patient to enroll to the study. Participants were withdrawn if, in the opinion of their GP, risks to their health were present. This could include a history of myocardial infarction, angina, stroke, and chronic pulmonary disease (COPD). Three of the original 47 applicants were advised to withdraw under GP advice. The remaining participants completed a physical activity readiness questionnaire (PAR‐Q) and provided written informed consent to participate in the study, which was approved by the University of the West of Scotland research ethics committee.

### Study participants

**Figure 1.**
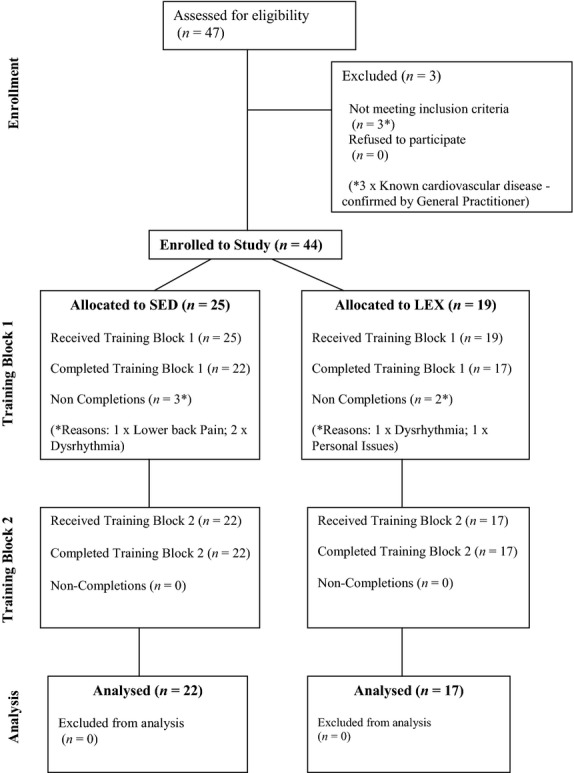
Flow diagram depicting transit of lifelong sedentary (SED) and lifelong exercising (LEX) participants through the study.

Forty‐four participants were enrolled to the study and allocated one of two distinct groups. Group one consisted of lifelong sedentary (SED) men (*n* = 25; aged 62.3 ± 4.6 years) who did not participate in any formal exercise program and had not done so for a minimum of 30 years. Group two consisted of male lifelong exercisers (LEX), (*n* = 19; aged 61.3 ± 5.1 years), who were highly active and completed on average 280 min of structured exercise training each week (range 180–550 min week^−1^). (12/19 of LEX were active masters national competitors in sports including triathlon, athletics, sprint cycling, and racquet sports). Figure 1 depicts the passage of participants through the study. Prior to physiological assessment, participants were familiarized with equipment and techniques that would be employed during the course of the study (Table 1).

**Table 1.** Participant characteristics for lifelong sedentary (SED) and lifelong exercising (LEX) aging males that enrolled to and completed the study. Data are presented as means (± SD)

	SED	LEX
Number of participants	22	17
Age (years)	62.7 ± 5.2	61.1 ± 5.4
Stature (cm)	175 ± 6.1	173 ± 5.5
Body Mass (kg)	89.9 ± 17.1	79.5 ± 12.3**
Body Mass Index (kg m^2^)	29.3 ± 5.0	26.4 ± 3.0**

** Denotes significant (*P *<**0.01) difference between groups.

### Study design

**Figure 2.**
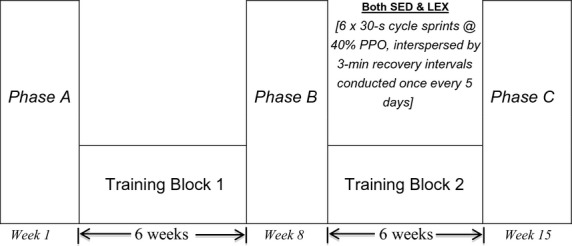
Schematic depicting study design incorporating three testing phases (A, B, and C) of two distinct training blocks for lifelong sedentary (SED) and lifelong exercising (LEX) groups.

The study employed a prospective cohort intervention design with LEX group acting as a positive control. As SED participants were unaccustomed to exercise and the effects of HIIT exercise in sedentary aging men is largely unknown, prudence dictated that prior to undertaking HIIT training SED should undertake 6 weeks of supervised progressive conditioning exercise (Training Block 1). This training block was designed to meet the ACSM guidelines of moderate‐intensity cardiorespiratory exercise training of 150 min week^−1^ (≥30 min day^−1^ on ≥5 day week^−1^). LEX maintained their normal exercise regimens. SED and LEX participants kept a weekly log detailing exercise achievements, which was documented and confirmed using telemetry data downloaded from HR monitors (Polar, Kempele, Finland) at the end of each week. Figure 2 outlines a schematic of study design, which consisted of three sample points. Phase A was baseline measurement; Phase B was conducted following training block 1, during week seven. Following Phase B, both groups undertook training block 2. This training block was targeted at performing low frequency, high‐intensity interval training (L_*f*_HIIT) performed once every 5 days (6 × 30‐s sprints at 50% peak power on a cycle ergometer interspersed with 3 min recovery intervals). This was the only exercise performed during this period and immediately preceded Phase C measurements in week 15. At each measurement phase, data were obtained between 72 and 108 h following the last exercise session.

Power calculation was based on previously published data regarding exercise‐induced differences in endurance trained and untrained older but otherwise healthy men (Rywik et al. 1999). Using an estimated effect size on 0.8, sample size was calculated using a single‐tailed within group comparisons with *α *= 0.05 and *β *= 0.8 resulting in a required sample size of 17 participants per group.

### Laboratory measures

On assessment phases A, B, and C, participants arrived in the exercise physiology laboratory between the hours of 07.00–09.00 am, following an overnight fast and having abstained from strenuous exercise for a minimum of 48 h. Participants were reminded to maintain standardized conditions prior to each assessment point which included arriving in a hydrated state having abstained from caffeine and alcohol consumption for 36 h. Following 20 min supine rest blood was sampled, from the nondominant arm using the standard venipuncture method into sterile serum separator vacuutainer tubes (Becton Dickinson, Rutherford, NJ) that were kept at room temperature in the dark, for 30 min, to allow for clotting, after which samples were centrifuged at 1100 g at 4°C for 15 min. Serum was then extracted, aliquoted and stored at −80°C until subsequent analysis. Blood samples were collected at the same time of day for each participant in an attempt to control for biological variation (Reilly and Brooks 1982) and minimize intersubject analytical variation.

### Flow mediated dilation

Subsequently and using the contralateral arm, endothelium‐dependent vascular responses of the brachial artery were assessed by high‐resolution ultrasound imaging and automated vessel‐diameter measurements (Charakida et al. 2010). Ultrasound images were recorded with an ATL HDI 3000CV Ultrasonography device (Advanced Technology Laboratories Inc., Washington) using an ATL L7‐4 (38 mm) 14 MHz linear array transducer. Participants were required to lay supine for 10 min prior to starting the measurement. Following which, a straight, nonbranching segment of the brachial artery above the antecubital fossa was identified and imaged in a longitudinal plane, with simultaneous capture of blood flow gated pulse wave Doppler imaging. The Doppler gate was set to encompass the majority of the width of the artery, and was angle corrected at 60^°^. Depth, gain, and zoom settings were adjusted to optimize image quality, and recorded for future image acquisition. Brachial artery diameter was recorded for 1 min (baseline) following which a cuff was then inflated to suprasystolic pressure (approx. 220 mmHg) on the upper forearm, distal to the imaging site for 5 min. At the end of 5 min the cuff was rapidly deflated and the segment of the brachial artery and blood flow was recorded continuously for another 5 min. R‐wave gated frames where not exclusively captured as data from our lab and others indicate no difference between R‐wave gating and images captured at four frames per second (Padilla et al. 2008). Brachial artery diameter was measured offline by an automatic edge‐detection system (Brachial Analyzer, Medical Imaging Applications LLC, Coralville). The same software also calculated blood flow using the envelope of the Doppler spectral traces which was subsequently used to calculate hyperemic shear. Shear data were exported to a spreadsheet and the area under the shear rate curve up to the point of maximal arterial dilation, was calculated based on the Reimann sum technique as previous described (Black et al. 2009) Change in vessel diameter was calculated using 3 sec averaging and expressed as percentage change from baseline. As FMD changes are partly dependent upon vessel diameter absolute diameter changes are also presented. The coefficient of variation (CV) for the FMD measurement in our laboratory is < 5.6%.

### Body composition

Body mass was obtained using a balanced weighing scales, with participants in minimal clothing (Seca, Cardiokinetics, Salford, UK) and height was measured with a stadiometer (Seca, Cardiokinetics, Salford, UK). Weighing scales were calibrated prior to each “weigh in” with a 5 kg free mass. Body Mass Index (BMI) was calculated by dividing subject weight in kilograms by the square of the participant's height in meters.

### Determination of maximal aerobic capacity 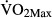


Aerobic capacity determined using open circuit spirometry via a Cortex II Metalyser 3B‐R2 (Cortex, Biophysik, Leipzig, Germany). Expiratory airflow was achieved using a volume transducer (Triple V^®^ turbine, digital). Expired gases were analyzed for O_2_ with electrochemical detection and for CO_2_ with an infrared analyzer. Prior to each test, the Metalyser was calibrated according to manufacturers' guidelines. After a 60 min warm‐up period, the CO_2_ and O_2_ sensors were calibrated against room air and to a reference gas of known composition (5% CO_2_, 15% O_2_, and 80% N_2_). Volume measurement was calibrated by five inspiratory and expiratory strokes using a 3‐L syringe. Five minutes of warm‐up exercise preceded a ramped protocol until volitional exhaustion on an air‐braked cycle ergometer (Wattbike Ltd., Nottingham, UK). Saddle height was adjusted relative to the crank position with participants knee joint at almost full extension (approx. 170–180°), and the foot was secured to a pedal with clips. Participant performance on a peak power test dictated the cadence (either 70, 75; 80; 85 rpm) to be maintained throughout the 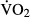 assessment. Peak power output (PPO) was determined using a 6 s peak power test on a Wattbike Pro cycle ergometer, which we have recently shown to be a valid measure of PPO generated during 30 s Wingate test on a Monark 818E cycle ergometer (Herbert et al. 2014). Participants warmed up at 100 watts at the cadence they would use in the test, which was conducted using a modified Storer Test (Storer et al. 1990). Work‐rate was increased by 18 W each minute until volitional exhaustion was achieved. Based on prior pilot study, the test was expected to elicit 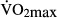 in 10 ± 2 min. Oxygen uptake 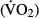, carbon dioxide production 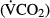 respiratory exchange ratio (RER), ventilation 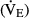 were displayed continuously. Heart Rate (HR) was recorded every 5 sec using short‐range telemetry (Polar T31, Kempele, Finland). Participants indicated perceived exertion using the Borg scale (Borg 1970) which was recorded during the last 10 sec of each 1 min stage. Fingertip blood lactate (BLa^−1^) samples were collected into a portable automated lactate analyser (Lactate Pro, Arkray, Inc., Kyoto, Japan) within 45 sec and again 5 min following the termination of the test. Breath by breath data were sampled and transferred to a PC for real‐time display. The recorded data were saved to the internal database (Metasoft version 3.7.0, Cortex Biophysik GmbH, Leipzig, Germany) until analysis.

### Training block 1

**Figure 3.**
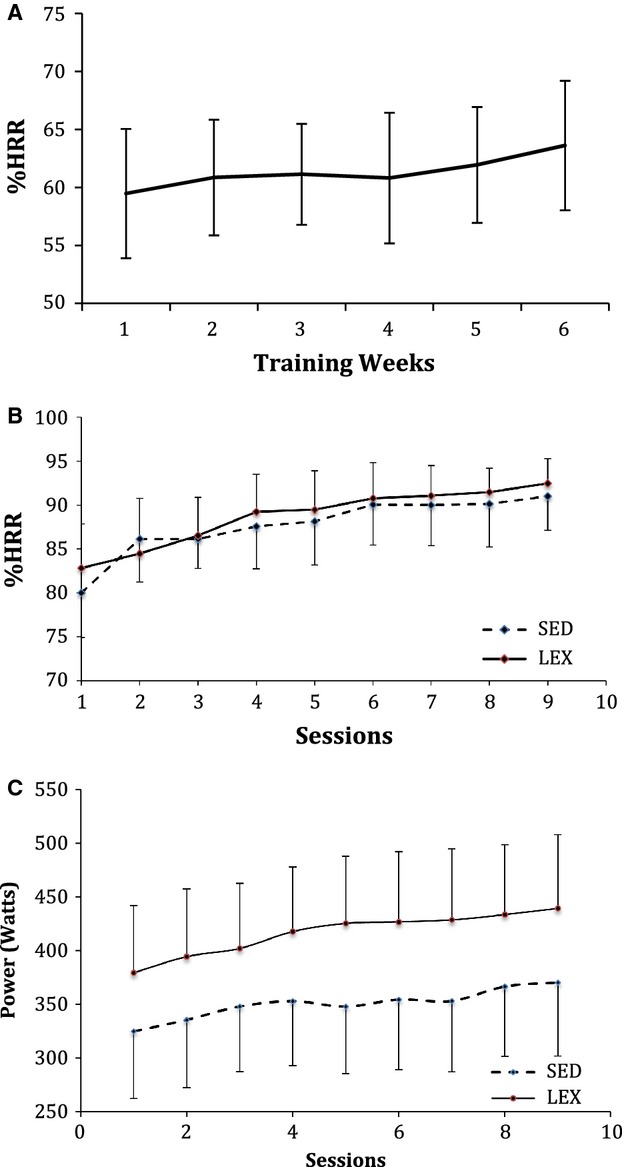
(A) Graph showing group mean weekly percentage heart rate reserve (%HRR) responses for lifelong sedentary men (SED) during exercise training in Training Block 1. (B) Depicts group mean %HRR for SED and a lifelong exercisers (LEX) during each of nine high intensity interval training (HIIT) sessions. Data are presented as mean ± SD. (C) Depicts group mean power output (MPO) for SED LEX during each of nine HIIT sessions. Data are presented as mean ± SD.

Sedentary participants underwent a 6‐weeks of personalized and supervised preconditioning exercise in accordance with the ACSM guidelines (Chodzko‐Zajko et al. 2009a) of 150 min week^−1^. Participants were familiarized with the use of a Polar FT1 heart rate monitors (Polar Team System, Polar Electro Oy, Kempele, Finland) enabling the recording of exercise time, average and peak heart rate during self monitored exercise sessions. The rate of progression intended to achieve an average heart rate reserve (HRR) of 55% during the first 2 weeks, increasing to 60% of HRR for the subsequent 2 weeks, with the final 2 weeks incorporating short bursts of higher intensity exercise into participant training sessions and elicit a HRR of 60–65%. Group mean weekly exercising %HRR for SED group is detailed in Figure 3A. Exercise training modes were optional, and included walking, walk/jogging, jogging, cycling, (flat terrain) cycling, (hill terrain), and adapted to suit the participants' physical status and personal preference. LEX participants were required to continue with their normal exercise training during training block 1. All participants were contacted weekly by e‐mail or telephone in order to monitor exercise type, frequency, intensity, and duration thereof.

### Training block 2

The rationale for extended recovery between HIIT sessions in training block 2 was supported by data obtained during a pilot study using masters athletes [(*n* = 10; mean age 63 ± 3.4 years)], where 3 and 5 day recovery strategies from single HIIT sessions (6 × 30 sec at 50% PPO) were compared and identified that 5 days of recovery allowed for optimal recovery between sessions to be achieved.

Training block 2 consisted of L_*f*_HIIT training performed once every 5 days for 6 weeks (nine sessions) with each session consisting of 6 × 30 sec sprints at 50% of peak power output determined during familiarization. Sessions were performed on Wattbike Pro cycle ergometers (Wattbike Ltd., Nottingham, UK) which was interspersed with 3 min active recovery intervals against a low (0–50 W) resistance and self‐selected speed (rpm). HIIT sessions were conducted in groups of between four and six participants.

The first three training sessions were used to familiarize the participants to high intensity exercise, working at 40% of their maximum peak power measured at Phase B. Subsequent sessions were conducted at 50% of predefined peak power output. Based on the same pilot study, the target power outputs (40%, 50%) were required to achieve peak heart rates >90% HRR during the 30 s efforts. Group mean peak and average %HRR's for SED and LEX during each HIIT session were recorded using a Polar Team System and software and are detailed in Figure 3B. Group mean power output, for SED and LEX during each HIIT session is detailed in Figure 3C. The HIIT sessions were the only exercise performed by both SED and LEX groups during this training block period and preceded Phase C measurement by between 72 and 108 h following the last HIIT session.

Serum concentration of VEGF was determined in duplicate, according to the manufacturer's guidelines, by an investigator blinded to the study using solid phase sandwich enzyme‐linked immunosorbent assay kits (Invitrogen Corp., Carlsbad, CA). The Hu VEGF standard was calibrated against a highly purified recombinant *SF*21 expressed Hu VEGF‐165 protein. The minimum detectable dose of VEGF was <5.0 pg/mL. The mean value of the two measures was used for the analyses. Inter‐ and intra‐assay CV's for the determination of VEGF were < 9.3% and < 5.5% respectively.

### Statistical analysis

Data were analysed using SPSS version 20.0 (IBM, Armonk, NY). Q‐Q plots were employed to confirm normal distribution of data. Training effects were compared using a 2 × 3 (group × time) mixed design ANOVA with pairwise comparisons of within group and between group simple main effects including a Bonferroni correction. An alpha value of *P *≤**0.05 was used to indicate statistical significance. Data are presented as mean ± standard deviation (S.D).

## Results

### Flow mediated dilatation

**Figure 4.**
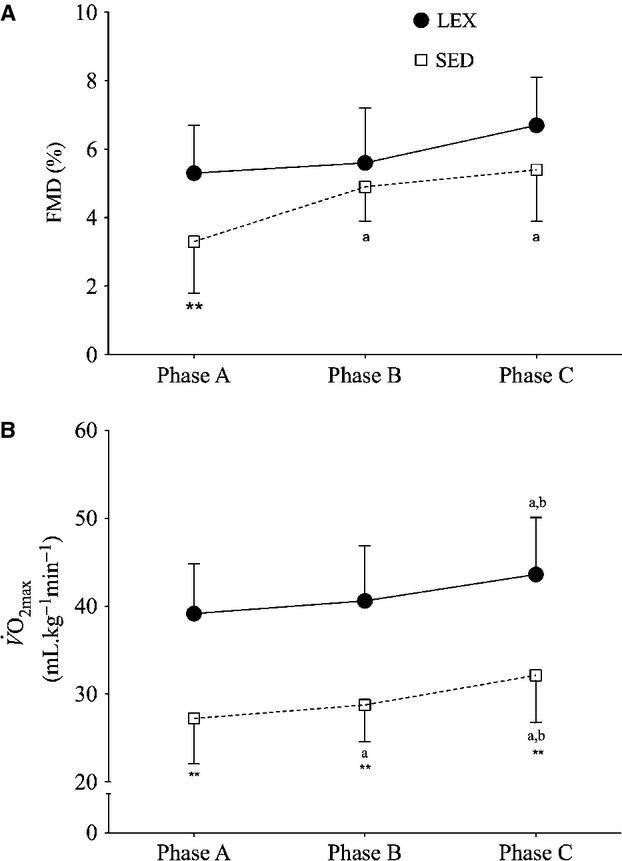
Changes in flow mediated dilatation (FMD: (A) and 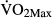 :(B) in lifelong sedentary (SED) and lifelong exercisers (LEX) on enrolment to the study (Phase A); following conditioning exercise (Phase B) and following low frequency high intensity exercise (L_*f*_HIIT; Phase C). Data are presented as mean ± SD. **P <**0.01 versus LEX and same time‐point, ^a^*P *<**0.01 versus Phase A in the same group, ^b^*P *<**0.01 versus Phase B in the same group.

Analysis revealed a significant difference in FMD between groups (*P *<**0.001) and a significant main effect of measurement phase (*P *<**0.001), however, there was no interaction between groups and measurement phase (*P = *0.193). FMD was lower in SED compared to LEX at Phase A (3.4 ± 1.5% vs. 5.4 ± 1.4%; *P < *0.01, 95% CI 0.96 – 3.4), but was not different between groups at Phase B (4.9 ± 1.1% vs. 5.5 ± 1.9%; *P *>**0.05, 95% CI −1.971–0.659) while there was a trend for a difference at Phase C (5.4 ± 1.4% vs. 6.7 ± 1.5%; *P *=**0.053, 95% CI −2.651–0.020). In SED, there was an increase in FMD from Phase A and Phase B (*P < *0.001 95% CI −2.333 to −0.741). There was no change in FMD between Phase B and Phase C (*P *=**1.0 95% CI −1.989–1.049). FMD did not change in LEX between any time points (*P *>**0.05 for A–B, B–C or A–C) (Fig. 4).

### Vascular endothelial growth factor

There was no main effect of Phase for vascular endothelial growth factor (VEGF) (*P *>**0.05), however, there was a significant effect of group (*P *<**0.01) and an interaction effect (*P *<**0.05). At Phase A VEGF was higher in SED (*P *<**0.01, 95% CI 44.25 – 193.45), however, there were no differences between groups at Phase B (*P *>**0.05, 95% CI −39.0–144.3) although VEGF was higher in SED at Phase C (*P *<**0.01, 95% CI 98.0 – 289.1). SED did not change from either Phase A to Phase B, or from Phase B to Phase C (both *P *>**0.05), but did increase between Phase A and C (*P *<**0.01, 95% CI 291.9 – 137.1). There were no changes in VEGF in LEX between any Phases (all *P *>**0.05) (Table 2).

**Table 2.** Determinants of vascular function and biomarkers of angiogenesis for lifelong sedentary and lifelong exercisers on enrolment to the study (Phase A); following conditioning exercise (Phase B) and following low frequency high intensity exercise (L_*f*_HIIT; Phase C). Data are presented as mean ± SD

	SED			LEX		
	Phase A	Phase B	Phase C	Phase A	Phase B	Phase C
Brachial Diameter (mm)	4.9 ± 0.05	4.8 ± 0.05	5.0 ± 0.06	5.1 ± 0.05	5.0 ± 0.06	5.0 ± 0.06
SR_AUC_ (*s* × 10^3^)	15.1 ± 4.0	14.7 ± 3.6	16.4 ± 4.3	15.5 ± 3.0	16.5 ± 2.9	16.0 ± 4.3
IGF‐I (ng mL^−1^)	13 ± 4.6*v	15 ± 5.8	17 ± 4.5Ω	18 ± 6.2	17 ± 4.8	17 ± 4.0
VEGF (ng mL^−1^)	248 ± 93**	256 ± 104	327 ± 120**^a^	129 ± 64	203 ± 97	134 ± 80

SR_AUC_, area under the shear rate curve to maximum dilatation; IGF‐I, Insulin like growth factor‐I; VEGF, vascular endothelial growth factor.

*P <**0.05 versus LEX at same time‐point.

** *P *<**0.01 versus LEX and same time‐point.

Ω *P *<**0.05 versus Phase A in the same group.

a *P *<**0.01 versus Phase A in the same group.

## Discussion

The main findings of the present study are that SED demonstrate inferior vascular function and aerobic capacity compared with LEX on enrolment to the study. Significant improvements in vascular function can be achieved in lifelong sedentary aging men following a period of structured progressive conditioning exercise and maintained but not further improved following a subsequent L_*f*_HIIT.

### Maximal aerobic capacity 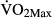


The observation that on enrolment LEX had ~44% higher 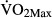 than SED is similar to recent comparisons of aerobic capacity in trained and untrained seniors [46%; (Iversen et al. 2011; Shibata and Levine 2012)]. It also supports the commonly reported perception that age related decrements in aerobic capacity are more pronounced amongst untrained individuals (McGavock et al. 2009; Seals 2014). Furthermore, SED demonstrated improvements following both conditioning exercise and L_*f*_HIIT supporting previous work demonstrating aerobic capacity recovery following exercise training in older adults (Poulin et al. 1992; Murias et al. 2010).

Following the conditioning exercise, SED achieved a small, but significant decrease in body mass, with a corresponding increase in relative 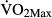 (~5.5%). This increase is of a lower magnitude than is commonly reported in studies of short duration (9–12 weeks) of aerobic exercise in healthy older males (Poulin et al. 1992; Beere et al. 1999; Gass et al. 2004; Murias et al. 2010). The larger magnitude of change reported in those studies may be due to the use of higher intensities (Poulin et al. 1992; Beere et al. 1999; Murias et al. 2010) or longer durations of intervention (Poulin et al. 1992; Beere et al. 1999; Gass et al. 2004). Additionally, since the first 2 weeks of conditioning exercise in the present study was at a lower intensity, to allow participants to adapt in a progressive manner to their increased physical activity, the time available for adaptation was further reduced.

In the present study, both LEX and SED demonstrated significant increments in maximal aerobic capacity following L_*f*_HIIT training, with mean increases of 7.4% and 8.3% in LEX and SED respectively. There is limited comparative data regarding the efficacy of reduced frequency of HIIT on aerobic capacity. Nakahara et al. (2014) using young healthy males demonstrated larger improvements in aerobic capacity than L_*f*_HIIT in the present study. Over a longer period (3 months) using one session per week consisting of three bouts at 80% maximum work rate to volitional exhaustion they demonstrated a 13% increase in 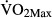. Similarly there is evidence that reducing the volume of sessions, but maintaining their frequency at 3 week^−1^ also does not adversely impact on the improvements in aerobic capacity (Matsuo et al. 2014; Zelt et al. 2014).

The present data are also comparable to previous studies examining standard frequency HIIT in healthy young adults. A recent meta‐analysis of 37 HIIT studies reported an average increase in 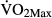 of 0.51 L min^−1^ (95% CI: 0.43–0.60 L min^−1^; (Bacon et al. 2013). This is comparable, although higher than that achieved in the present study (mean absolute increase = 0.397 L min^−1^ 95% CI: 0.24–0.47 L min^−1^ following L_*f*_HIIT in SED). The meta‐analysis only considered studies of young sedentary/moderately active participants (*n* = 334) under the age of 45 years, engaging in HIIT training a minimum of three times per week for durations of between 6 and 13 weeks and incorporating a minimum of 10 min of high intensity work per session interspersed with a minimum of 1:1 recovery intervals. The larger mean increase reported by Bacon et al. (2013) may be due to differences in the duration of intervention. L_*f*_HIIT in the present study was implemented for 6 weeks. Inclusion criteria for the meta‐analysis required interventions to last a minimum of 6 weeks. Additionally, the difference may be a reflection of the greater scope for increased 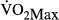 in younger cohorts. More recent studies have also reported similar increases in aerobic capacity in response to HIIT (Tjonna et al. 2013) and SIT (Macpherson et al. 2011) in younger adults.

### Vascular function

Neither maintaining their regular exercise, nor changing to L_*f*_HIIT improved vascular function in LEX. Given that LEX presented with superior FMD on enrolment to the study, it is likely they had limited scope for further improvements and is in line with the majority of findings in master athletes and physically active older men (DeVan and Seals 2012).

Following conditioning exercise, SED demonstrated significant improvements in FMD such that any prior difference between SED and LEX was diminished, signifying a complete recovery of vascular function. Additionally it is interesting to note, that the recovery of FMD occurred in the absence of any significant increase in absolute 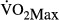, although relative aerobic capacity was increased (~5.5%) through reductions in body mass. Recovery of brachial FMD in healthy aged individuals undertaking moderate intensity endurance training has been reported previously (Pierce et al. 2011; Suboc et al. 2014), although not universally demonstrated (Black et al. 2009). Studies that have found aerobic exercise to improve vascular function have used lower intensities (e.g. daily walking and longer durations of 8 and 12 weeks respectively; (Pierce et al. 2011; Suboc et al. 2014)) than used by SED during the conditioning exercise. The present findings add to the literature by indicating that brachial FMD can be improved within 6 weeks in lifelong sedentary aging men. The improvement in FMD in a shorter timeframe than has previously been reported may be related to the higher mean intensity during supervised conditioning exercise compared to previous studies in healthy aging males (Black et al. 2009; Pierce et al. 2011; Suboc et al. 2014).

Given that following conditioning exercise, vascular function had recovered in SED to the extent that they no longer differed to LEX, there was little scope for further improvements in FMD following L_*f*_HIIT. Previous work has demonstrated significant reversal of brachial FMD following 2 weeks of detraining in a blood flow restricted exercise model, (Hunt et al. 2012) and within 4 weeks of detraining in patients with previous myocardial infarction (Vona et al. 2009). Correspondingly, the present data also adds to the literature by demonstrating that despite the low frequency of exercise and the low total effort time, L_*f*_HIIT provided sufficient stimulus to maintain the recovery of vascular function.

Age related vascular dysfunction is considered to be mediated in part by interactions between low‐grade inflammation and concomitant oxidative stress (Seals 2014). Aerobic exercise has been suggested to have direct protective effect reducing the deleterious effects of both superoxide associated vascular damage (Eskurza et al. 2004a,b) and low grade inflammation (Lesniewski et al. 2011). Consequently, regular exercise is particularly important for maintaining vascular health with advancing age (Seals 2014).

### Vascular endothelial growth factor and insulin like growth factor‐I

The present study revealed a significant group by time interaction for serum VEGF which was significantly higher in SED both on enrolment to the study and following L_*f*_HIIT (both *P *<**0.01) and increased significantly from enrolment to the completion of L_*f*_HIIT (*P *<**0.05). IGF‐I also demonstrated an interaction effect that was significantly lower in SED on enrolment to the study (*P *<**0.01) and increased between enrolment to the study and completion of L_*f*_HIIT. There is convincing evidence that vascular endothelial growth factor (VEGF) plays a mediating role in the process of angiogenesis (Prior et al. 2004) by promoting vasculogenesis, modulating the bioavailability of nitric oxide (Swift and Weinstein 2009) and encouraging the activation of progenerator cells (Yla‐Herttuala et al. 2007). Additionally, a growing body of evidence suggests that reduced GH and IGF‐I levels are causally related to vascular aging (Bailey‐Downs et al. 2012).

IGF‐I has also been shown to mediate the upregulation of VEGF in the rodent (Lopez‐Lopez et al. 2004) and more recently, in embryonic stem cell models (Piecewicz et al. 2012). This upregulation requires the transcriptional modulator hypoxia‐inducible factor (HIF)‐1*α*, which is highly sensitive to local oxygen tension (Maxwell and Ratcliffe 2002). In the present study, FMD improved in SED prior to any changes in IGF‐I or VEGF. The lack of similar changes in LEX may indicate that existing vascular function and capillarization were sufficient to meet the metabolic demands of the L_*f*_HIIT protocol. This suggests that in older, sedentary but otherwise healthy males, recovery of vascular dysfunction occurs prior to increases in markers of angiogenesis. Alternatively, given the role of HIF‐1*α*, the protracted appearance of increases in VEGF and IGF‐I may be due to L_*f*_HIIT inducing greater local oxygen demand and consequently HIF‐1*α* mediating greater increases in VEGF. Irrespective of mechanism, in the present study IGF‐I and VEGF continue to increase beyond the point at which FMD plateaus and suggests that the L_*f*_HIIT stimulus was sufficient to drive further angiogenic signals. Evidence from masters athletes demonstrates that they enjoy greater capillarization than their sedentary counterparts (Iversen et al. 2011) which may go some way to explaining the interaction effects for IGF‐I and VEGF in the present study. The large intraindividual variability in VEGF (≈37–50% on enrolment) makes conclusions from direct comparisons between SED and LEX difficult. However, the significant interaction effects for FMD, VEGF, and IGF‐I suggest that all three share convergent mechanisms for the initiation of vascular remodeling.

The preponderance of work investigating the potential for VEGF to be influenced by exercise has involved patients with existing peripheral arterial and coronary artery disease and have reported increases (Adams et al. 2004; Sandri et al. 2005, 2011; Park et al. 2010) or lack of change (Danzig et al. 2010; Schlager et al. 2011; Beck et al. 2012; Voss et al. 2013) in response to exercise. As with studies of FMD and aerobic capacity in older participants with existing disease, the underlying pathology and ongoing pharmacological intervention limit comparisons with healthy sedentary aged participants. The only previous examination of exercise and VEGF in sedentary healthy older adults used 1 year of walking and failed to demonstrate any changes (Voss et al. 2013). However, the extended time frame, lower intensity and a predominantly postmenopausal cohort, again limits comparisons with the present study.

The present study has some important limitations that should be noted. One concerns the proximity of the conditioning program (training block 1) to the L_*f*_HIIT intervention (training block 2), which makes it impossible to rule out the contribution of conditioning exercise to the overall effect on SED following L_*f*_HIIT. At the time of writing, the effects of HIIT on in older sedentary participants is currently unknown, and the authors deemed it prudent to gradually prepare the SED cohort by introducing them to supervised progressive exercise training. This is further justified when one considers the 100% adherence to the L_*f*_HIIT subsequent to cardiovascular conditioning. Furthermore, aerobic improvements in LEX in response to L_*f*_HIIT indicate that similar improvements in SED are as a consequence of the L_*f*_HIIT stimulus, rather than a residual effect of the conditioning exercise. A further limitation is the absence of a negative control group, particularly between Phases B and C. Correspondingly it is not possible to confirm that L_*f*_HIIT *per se* was responsible for the maintenance of vascular function. Additionally, the present study was powered to identify changes in 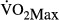. Given the wide variability of VEGF and IGF‐I, the study is underpowered to identify changes in these parameters. Consequently, the possibility that significant changes have been missed cannot be discounted.

In conclusion, although L_*f*_HIIT is an effective training modality to increase cardiorespiratory fitness in both lifelong sedentary and lifelong exercising aging men but 6 weeks of L_*f*_HIIT does not improve FMD beyond that achieved in conditioning exercise, however, the stimulus is sufficient to maintain improvements in vascular function and influence markers of angiogenesis.

## Conflict of Interest

None declared.
